# Sanzi Sijun Formula Alleviates Lipotoxic Liver Injury in Metabolic Dysfunction-Associated Steatotic Liver Disease via AMPK/SIRT1 Signaling Pathway

**DOI:** 10.3390/ph19081195

**Published:** 2026-07-29

**Authors:** Junyao Ding, Tao Liu, Ping Huang, Lili Yang, Zhiwei Chen, Yining Xue, Yunlong Hua, Haiyan Song, Peiyong Zheng

**Affiliations:** Institute of Digestive Diseases, Shanghai University of Traditional Chinese Medicine, Shanghai 200032, China; dingjyo@163.com (J.D.); lh2488@126.com (T.L.); Hpingwork@163.com (P.H.); yanglili76@126.com (L.Y.); chenzhiweicn@126.com (Z.C.); xue3423@126.com (Y.X.); ri_cardo_l@live.com (Y.H.)

**Keywords:** metabolic dysfunction-associated steatotic liver disease, Sanzi Sijun Formula, AMP-activated protein kinase, silent information regulator 1, lipid metabolism disorder

## Abstract

**Objective:** While Sanzi Sijun Formula (SSF) has exhibited preliminary efficacy against metabolic dysfunction-associated steatotic liver disease (MASLD), its mode of action remains undefined. This study therefore aimed to unravel its core therapeutic mechanisms. **Methods:** UPLC-MS was employed to characterize the major components of SSF. Male C57BL/6J mice were fed a high-fat diet combined with high-fructose/glucose drinking water (HFD-HF/G) for 10 weeks to establish a MASLD model, followed by SSF intervention. After 8-week treatment, body and liver weight, hepatic histopathological alterations, serum levels of lipids, transaminase, and inflammatory cytokines were detected, and transcriptomic sequencing was performed on mouse liver tissues for mechanistic exploration. AML12 hepatocytes stimulated with palmitic acid (PA) were treated with SSF alone or in combination with AMPK or SIRT1 specific inhibitors. RT-qPCR and Western blotting were used to detect the expression or activation levels of AMPK, SIRT1, and key lipid metabolism-related molecules. **Results:** A total of 77 active components were identified in SSF by UPLC-MS analysis. In MASLD model mice, SSF significantly reduced body and liver weight, serum levels of total cholesterol (TC), triglyceride (TG), low-density lipoprotein cholesterol (LDL-c), and alanine aminotransferase (ALT), suppressed the pro-inflammatory cytokines including TNF-α and IL-6, and elevated adiponectin levels. Histopathological staining demonstrated that SSF effectively alleviated hepatic steatosis, ballooning, and inflammatory cell infiltration. Transcriptomic profiling analysis verified the major regulatory effect of SSF on lipid metabolism and identified the AMPK/SIRT1 signaling pathway as a potential mechanism. Further experiments confirmed that SSF restored the levels of AMPK/ACC phosphorylation and SIRT1 expression, thereby modulating downstream lipid metabolism-related genes in liver tissues. In PA-induced AML12 cells, SSF significantly reduced intracellular accumulation of lipid and reactive oxygen species (ROS), which were partially abrogated by the inhibitors of AMPK or SIRT1. **Conclusions:** SSF exerts prominent effects against MASLD in both in vivo and in vitro models. Modulation of the AMPK/SIRT1 signaling pathway primarily contributes to its therapeutic mechanism against lipid metabolism disorder and lipotoxic liver injury. These findings provide experimental evidence to support the clinical application of SSF for MASLD treatment.

## 1. Introduction

Nonalcoholic fatty liver disease (NAFLD) is a metabolic stress-induced liver injury that is closely associated with insulin resistance and genetic susceptibility and is characterized histopathologically by excessive hepatic lipid accumulation [1]. In 2023, a multi-society consensus statement renamed it MASLD to more accurately reflect its pathophysiological features [2]. The disease can progress from an early stage of simple hepatic steatosis, termed metabolic dysfunction-associated steatotic liver (MASL), to a more severe phase called metabolic dysfunction-associated steatohepatitis (MASH), and a subset of MASH patients further progress to cirrhosis or liver cancer [3]. With the growing epidemic of metabolic disorders, including obesity and type 2 diabetes mellitus (T2DM), the global prevalence of MASLD has now surpassed 30% [4]. Although dietary restriction combined with exercise can alleviate or even reverse MASLD, it is difficult for most patients to maintain long-term weight loss [5]. For patients at the MASH stage, the need for pharmacological treatment, to halt or reverse disease progression, is particularly urgent. In March 2024, the FDA granted accelerated approval to resmetirom, making it the first drug approved for MASH patients with moderate-to-advanced fibrosis [6]. Subsequently, in August 2025, semaglutide received accelerated FDA approval for the treatment of MASH with moderate-to-advanced fibrosis (F2-F3), based on the phase 3 ESSENCE trial [7]. Despite these breakthroughs, limitations in long-term safety, efficacy, and affordability restrict their widespread clinical use, highlighting the need for more accessible and safe alternatives.

To date, the mechanisms underlying MASLD progression remain incompletely understood, and involve a complex interplay of multiple factors, including lipid metabolic disorders, insulin resistance (IR), oxidative stress, and endoplasmic reticulum (ER) stress. Excessive hepatic lipid accumulation results from the overconsumption of dietary fat together with increased synthesis and decreased oxidation of fatty acids [8]. IR is a key driver in the pathogenesis of both MASH and T2DM [9]. It weakens the anti-lipolytic effect of insulin on adipose tissue, leading to the massive release of free fatty acids (FFAs) that are ectopically deposited in liver and other organs. Overwhelming β-oxidation of FFAs causes mitochondrial dysfunction, which in turn promotes oxidative stress [10]. Furthermore, increased lipid accumulation triggers the production of lipotoxic substances, leading to liver injury, inflammatory infiltration, and the activation of hepatic stellate cells, driving the disease progression [11].Therefore, reversing hepatic lipid metabolic disorders is an effective strategy for managing MASLD.

Traditional Chinese Medicine (TCM), characterized by its multi-target regulatory properties, has demonstrated notable clinical efficacy in treating MASLD in recent years. According to TCM theory, the core pathogenesis of MASLD is rooted in Spleen Deficiency, with Phlegm-Dampness and Stasis acting as the primary pathological factors [12]. Sanzi Sijun Formula (SSF) is a composite formula derived from two classic TCM prescriptions: Sanzi Yangqin Decoction, known for resolving phlegm and regulating qi [13], and Sijunzi Decoction, a representative formula for strengthening the spleen and replenishing qi [14]. This composition corresponds precisely to the most common syndrome of MASLD-Spleen Deficiency with Phlegm-Dampness Obstruction [15]. SSF consists of seven herbal medicines: *Ginseng Radix et Rhizoma*, *Atractylodis Macrocephalae Rhizoma*, *Poria*, *Glycyrrhizae Radix et Rhizoma*, *Sinapis Semen*, *Perillae Fructus* and *Raphani Semen*. Our previous studies confirmed that SSF could improve MASLD in mice at the MASL or MASH stage, ameliorating hepatic steatosis, inflammation, and insulin resistance [16]. However, its underlying mechanism remains unclear.

Therefore, using a high-fat diet combined with high-fructose/glucose drinking water (HFD-HF/G)-induced MASLD mice models, PA-induced AML12 cells, and RNA sequencing, this study aimed to explore the therapeutic mechanism of SSF against MASLD, which may benefit its clinical application. During the study, the RNA-seq data from liver tissues showed that the most significantly enriched pathways among the differentially expressed genes (DEGs) between MASLD mice with and without SSF treatment were related to lipid metabolism, such as fatty acid biosynthesis, the PPAR signaling pathway, fat digestion and absorption, and the AMP-activated protein kinase (AMPK) signaling pathway. Additionally, using network pharmacology, we have previously predicted that SSF could influence lipid metabolism disorders and inflammatory response pathways by targeting key molecules such as AKT, silent information regulator 1 (SIRT1), STAT3, and PPARG [17]. Both AMPK and SIRT1 are regarded as key metabolic regulators. Activated by sensing cellular energy status, AMPK can promote fatty acid oxidation while suppressing lipid synthesis [18,19]. Similarly, SIRT1 enhances mitochondrial function by activating peroxisome proliferator-activated receptor gamma coactivator 1-alpha (PGC-1α) through deacetylation, downregulating lipid synthesis via inhibiting sterol regulatory element-binding protein 1-c (SREBP-1c) expression, and inducing autophagy to clear lipid droplets [20,21]. AMPK and SIRT1 can synergistically regulate lipid metabolism, inflammatory responses, and fibrosis progression in MASLD. Therefore, combining our RNA-seq results with the prior network pharmacology analysis [17], we concentrated further mechanistic study on the AMPK/SIRT1 signaling pathway.

## 2. Results

### 2.1. The Major Components of SSF

**Figure 1:**
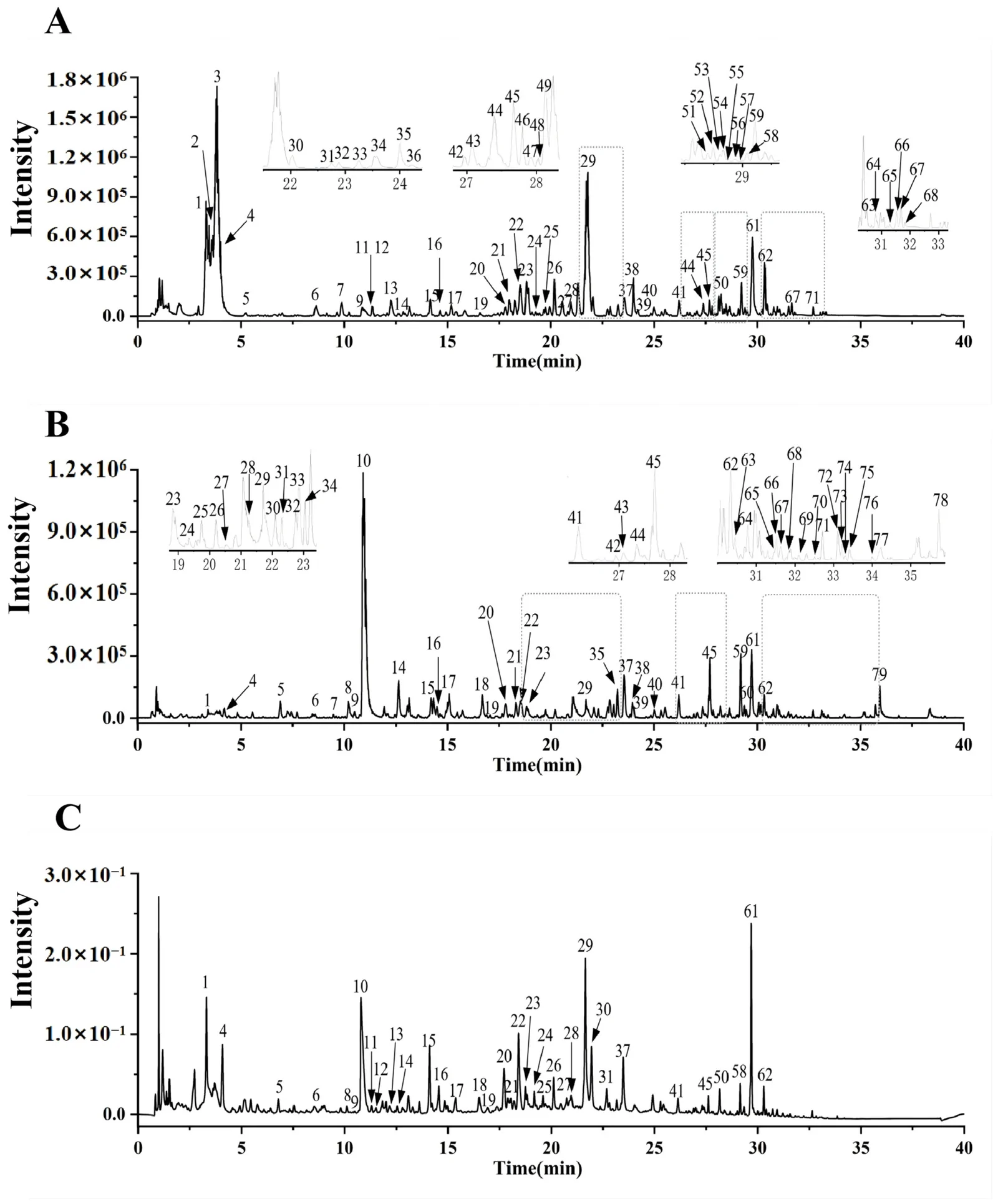
UPLC-MS analysis of the components of SSF. (**A**) UPLC-HRMS base peak chromatogram (BPC) of the SSF compound sample in negative ion mode. (**B**) UPLC-HRMS base peak chromatogram (BPC) of the SSF compound sample in in positive ion mode. (**C**) UPLC UV chromatogram of the SSF compound sample at UV 254 nm.

Using UPLC-MS analysis, 77 active chemical compounds and 2 packaging-derived artifacts were identified in SSF formulation (Figure 1, Table 1). Among the characteristic components, glucoraphenin, sinigrin, sinapine, and sinapic acid were from *Sinapis Semen* (Bai Jie Zi) and *Raphani Semen* (Lai Fu Zi); apigenin 7-O-β-D-glucuronide, rosmarinic acid, caffeic acid, and luteolin were from *Perillae Fructus* (Zi Su Zi); ginsenoside Rg1, Rb1 and Rf were from *Ginseng Radix et Rhizoma* (Ren Shen); atractylenolide II and III were from *Atractylodis Macrocephalae Rhizoma* (Bai Zhu); 6α-Hydroxypolyporenic acid C and Poricoic acid A, B and G were from *Poria* (Fu Ling); and liquiritin, isoliquiritin and glycyrrhizic acid were from *Glycyrrhizae Radix et Rhizoma* (Gan Cao). Additionally, quantitative analysis determined the contents of four major components in SSF: sinapine (3.87 mg/g), sinapic acid (2 mg/g), ginsenoside Rg1 (0.76 mg/g), and glycyrrhizic acid (1.82 mg/g) (Table 2, Supplementary Figure S1).

### 2.2. SSF Attenuated HFD-HF/G-Induced MASLD in Mice

**Figure 2:**
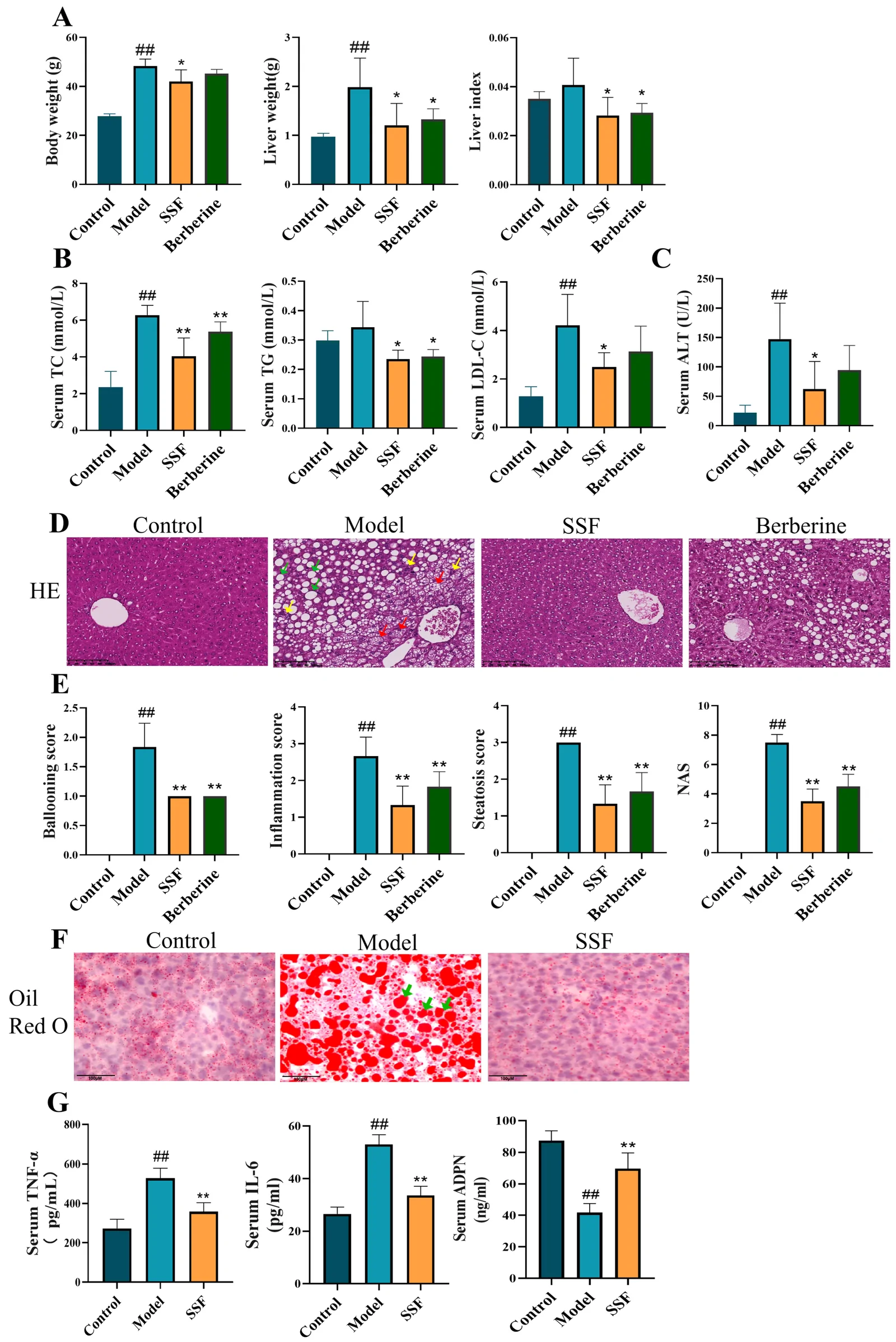
SSF ameliorated MASLD in HFD-HF/G-fed mice. (**A**) Body weight, liver weight, and liver index. (**B**) Serum levels of TC, TG, and LDL-c. (**C**) Serum ALT level. (**D**) H&E staining of mice liver sections. Arrows mark lipid droplets (green), hepatocyte ballooning (red), and inflammatory infiltration foci (yellow). Scale bar = 100 μm. (**E**) MAS assessment of mice liver sections. (**F**) Oil Red O staining of mice liver sections. Green arrows indicate lipid droplets. Scale bar = 100 μm. (**G**) Serum concentrations of TNF-α, IL-6 and ADPN. *n* = 6. ^##^ *p* < 0.01 vs. control; * *p* < 0.05, ** *p* < 0.01 vs. model.

HFD-HF/G-fed mice exhibited significantly greater body and liver weight than controls, both of which were markedly reduced by SSF intervention. In the berberine group, liver weight and liver index were also decreased (Figure 2A). Serum total cholesterol (TC), triglyceride (TG), low-density lipoprotein cholesterol (LDL-c), and alanine aminotransferase (ALT) levels were significantly elevated in the model group, and were effectively lowered by SSF. Berberine exhibited a modest downregulatory effect only on TC and TG levels (Figure 2B,C). H&E staining revealed extensive lipid droplet accumulation, hepatic steatosis, focal inflammatory infiltration, and hepatocyte ballooning in model mice, all of which were significantly ameliorated by SSF (Figure 2D). The MAS score exceeded 5 in the model group (indicative of MASH) and was notably reduced after SSF treatment (Figure 2E), whereas berberine produced a less pronounced histological benefit than SSF. Oil Red O staining further confirmed that SSF attenuated hepatic lipid accumulation. In addition, SSF markedly reversed the aberrant serum levels of IL-6, TNF-α, and ADPN (Figure 2F,G). Collectively, these results indicate that SSF effectively ameliorates HFD-HF/G-induced lipid metabolic dysfunction and lipotoxic liver injury in MASLD mice, with overall superior efficacy to berberine.

### 2.3. Transcriptomic Analysis of Liver Tissues Revealed the Key Regulatory Effect of SSF on Lipid Disorders

**Figure 3:**
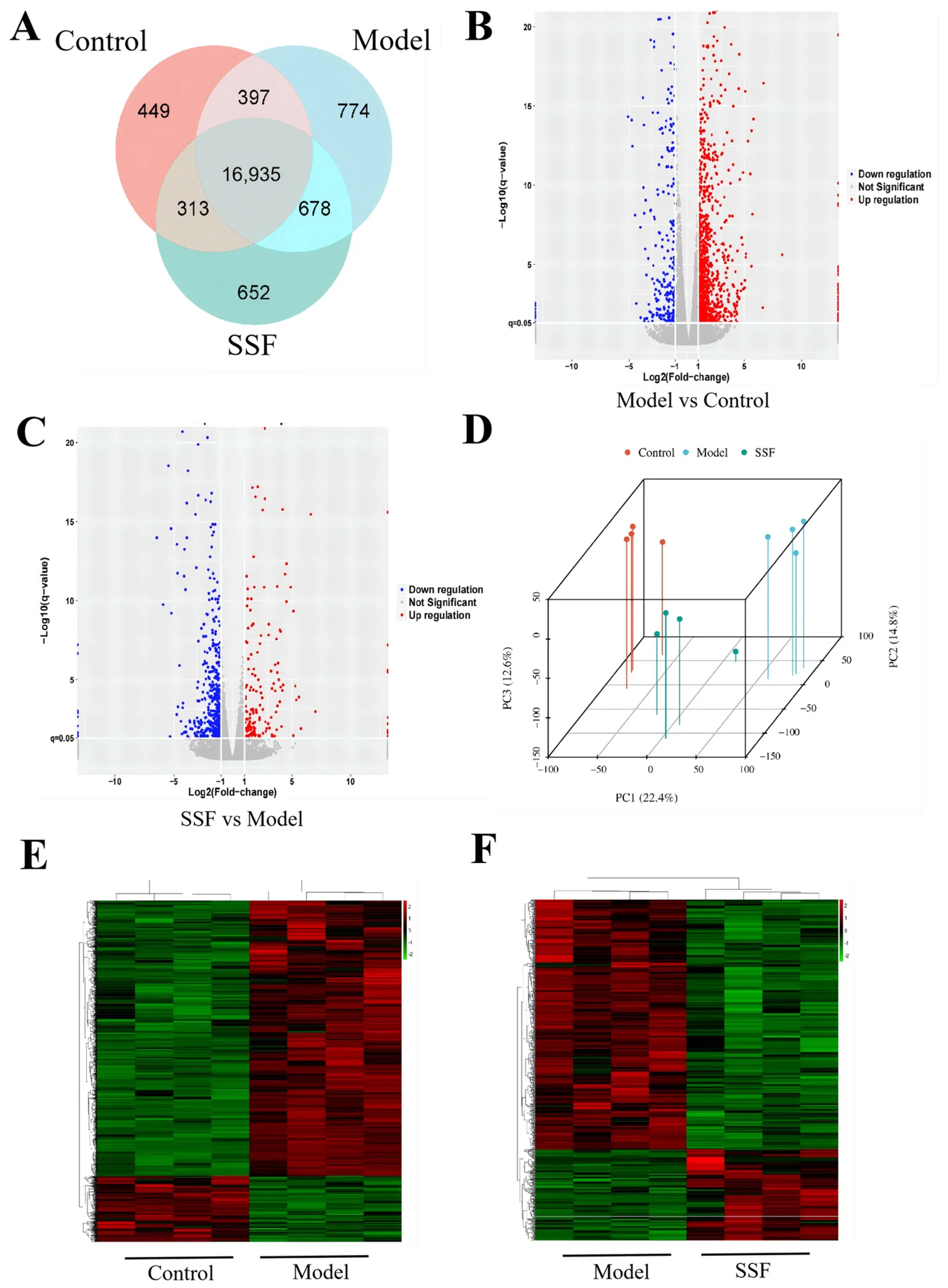
Transcriptomic analysis of differential gene expression profiles in liver tissues among the different mice groups. (**A**) Venn diagram. (**B**) Volcano plot of DEGs between the model and control groups. (**C**) Volcano plot of DEGs between the model and SSF groups. (**D**) PCA. (**E**) Clustering heatmap of DEGs between the model and control groups. (**F**) Heatmap of DEGs between the model and SSF groups. *n* = 4.

**Figure 4:**
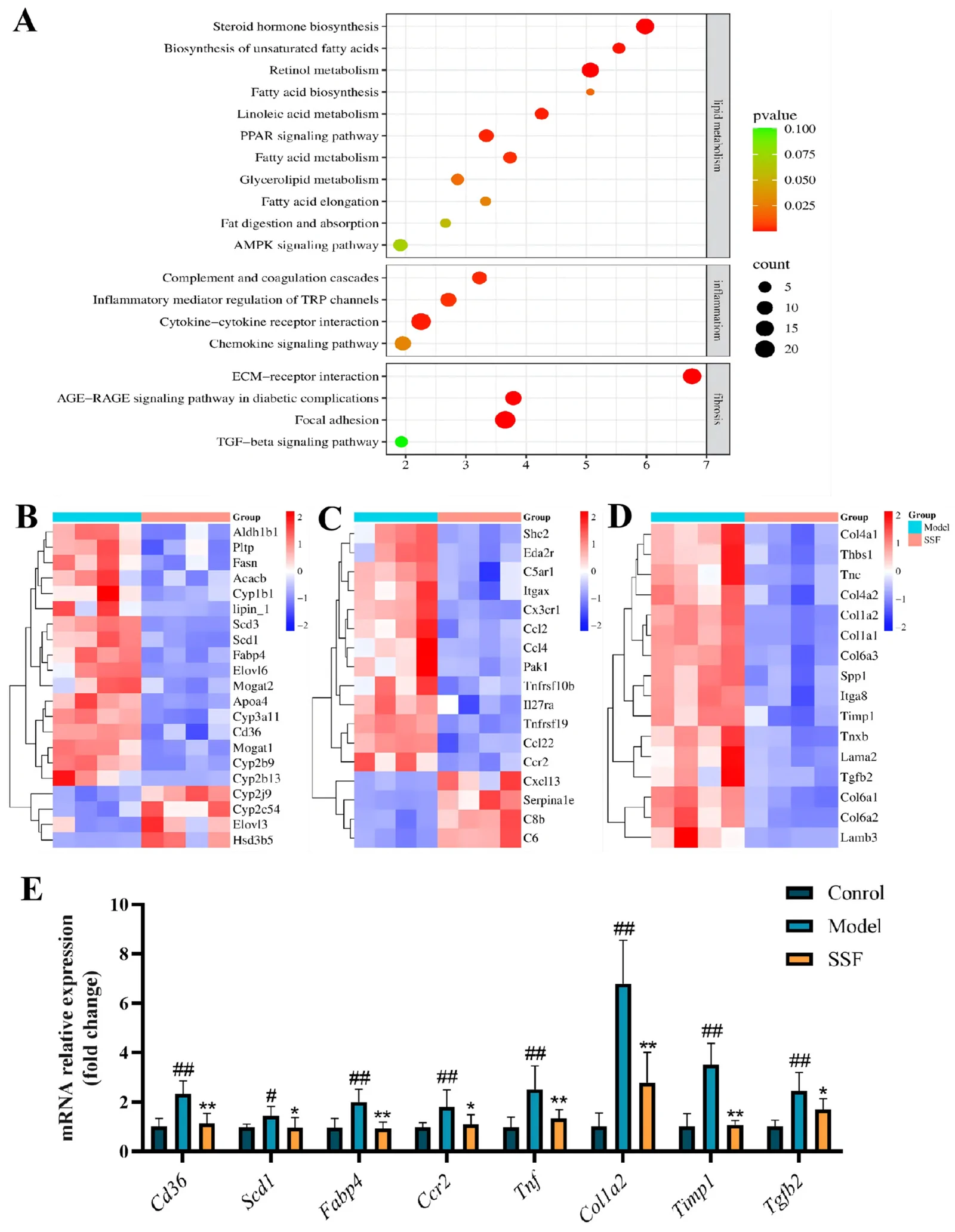
Transcriptomic analysis revealed multiple hepatic targets of SSF in MASLD mice. (**A**) KEGG pathway enrichment of DEGs. (**B**–**D**) Expression-level heatmaps of DEGs (SSF vs. model) associated with lipid metabolism, inflammation, and fibrosis. (**E**) The mRNA expression level of representative genes involved in lipid metabolism, inflammation, and fibrosis, measured by RT-qPCR. *n* = 4. ^#^ *p* < 0.05, ^##^ *p* < 0.01 vs. control; * *p* < 0.05, ** *p* < 0.01 vs. model.

To further elucidate the function and mechanism of SSF, we performed RNA sequencing of the mice liver tissues. Venn analysis visualized the unique gene transcriptional patterns shaped by diet and SSF treatment (Figure 3A). Volcano plots and heatmaps revealed numerous differentially expressed genes (DEGs) among the control, model, and SSF-treated groups. Compared with the control group, the model group exhibited 1377 DEGs, including 1110 upregulated genes and 267 downregulated genes. There were 602 DEGs present in the SSF group when compared to the model group, including 164 upregulated genes and 438 downregulated genes (Figure 3B,C). The principal component analysis (PCA) plot and the clustering heatmap of DEGs showed that the control, model and SSF samples formed distinct clusters, with significant inter-group differences in transcriptional profiles (Figure 3D–F). KEGG enrichment analysis of the DEGs (SSF vs. model) showed that the enriched pathways were mainly related to lipid metabolism, including fatty acid biosynthesis, the PPAR signaling pathway, fat digestion and absorption and the AMPK signaling pathway, among others. Additionally, pathways associated with inflammation and fibrosis were also found to be significantly enriched (Figure 4A). The expression patterns of representative DEGs from these pathways are visualized in the heatmaps in Figure 4B–D. Furthermore, some of the related genes were validated by RT-qPCR. As shown in Figure 4E, the expression levels of genes that promote lipid accumulation (*Cd36*, *Scd1*, *Fabp4*), inflammation (*Ccr2*, *Tnf*) and fibrosis (*Col1a2*, *Timp1*, *Tgfb2*) were increased in the liver tissues of model mice and were significantly downregulated by SSF. Collectively, these findings demonstrate that SSF exerts a multi-targeted reversal effect on the abnormal hepatic gene expression of MASLD mice, particularly involving genes related to lipid metabolism pathways.

### 2.4. SSF Activated the AMPK/SIRT1 Signaling Pathway and Improved Hepatic Lipid Metabolism in MASLD Mice

**Figure 5:**
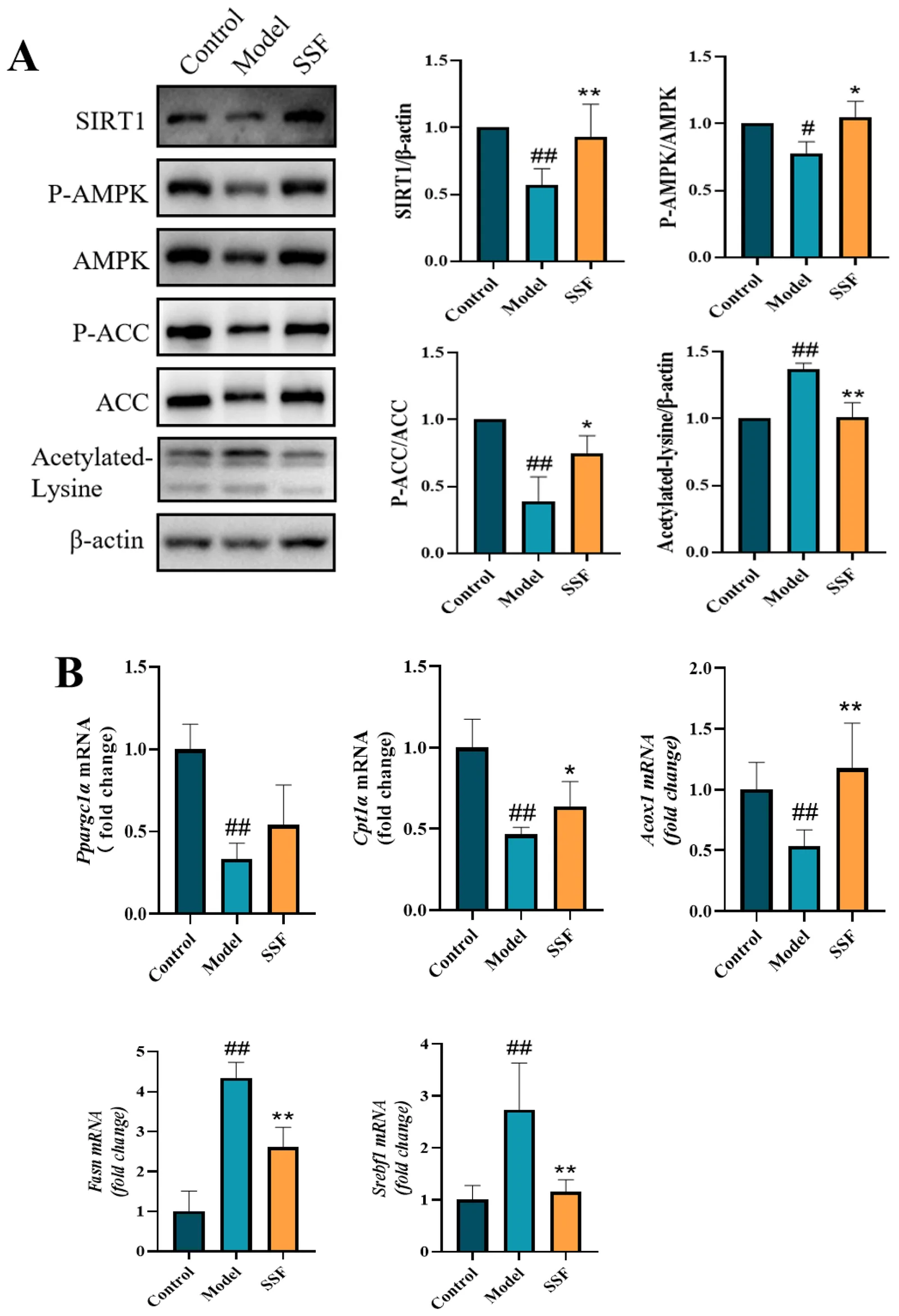
Effect of SSF on the AMPK/SIRT1 signaling pathway and lipid metabolism in MASLD mice. (**A**) Protein expression and/or activation level of SIRT1, AMPK, ACC and acetylated-lysine in mouse liver tissues. (**B**) mRNA expression levels of lipid metabolism-related genes in mouse liver tissues. *n* = 3–6, ^#^ *p* < 0.05, ^##^ *p* < 0.01 vs. control; * *p* < 0.05, ** *p* < 0.01 vs. model.

Given that hepatic transcriptomics revealed that SSF primarily regulates lipid metabolism pathways, including AMPK, and that our preliminary network pharmacology analysis predicted SIRT1 as its potential therapeutic target, we examined the regulatory effect of SSF on the AMPK/SIRT1 signaling pathway. Western blotting was performed to evaluate the expression or activation of key proteins in this pathway in mouse liver tissues. Compared with the control group, the model group exhibited significantly decreased phosphorylation of AMPK and its downstream target ACC, as well as reduced protein expression of SIRT1, all of which were effectively reversed by SSF intervention. As SIRT1 belongs to the family of deacetylases, quantification of acetylated-lysine levels serves as an indicator of its activation. In this study, SSF downregulated the elevated level of acetylated-lysine observed in the model group (Figure 5A). Meanwhile, we further examined the mRNA expression of downstream targets of the AMPK/SIRT1 pathway. As shown in Figure 5B, the expression of the lipid synthesis-related genes *Fasn* and *Srebf1* was downregulated by SSF treatment, whereas SSF upregulated the mRNA expression of key genes involved in fatty acid oxidation, such as *Ppargc1α*, *Cpt1α* and *Acox1*.

### 2.5. SSF Alleviated PA-Induced Lipid Accumulation and Oxidative Stress in Hepatocytes by Activating the AMPK/SIRT1 Signaling Pathway

**Figure 6:**
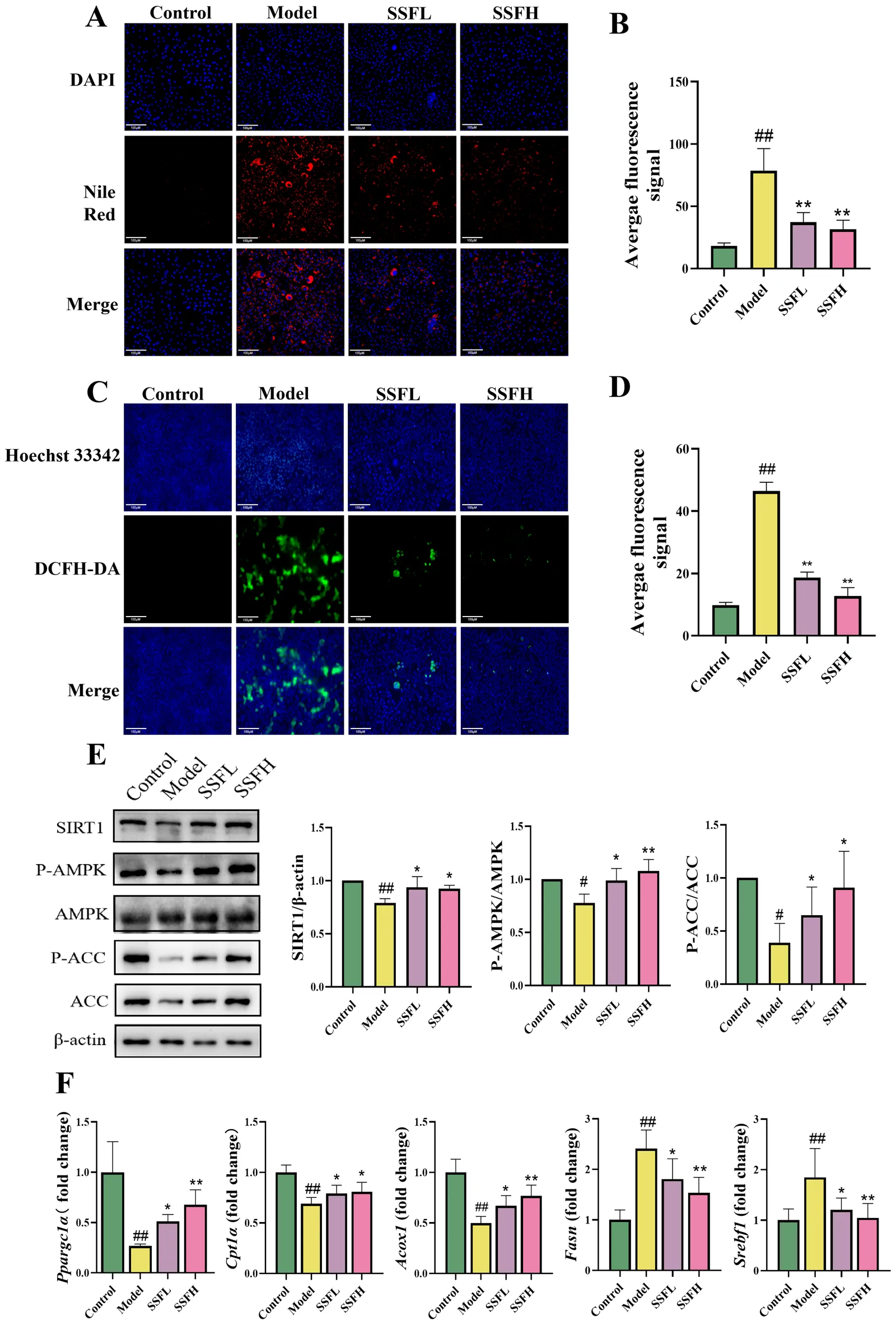
SSF mitigated PA-induced lipid accumulation and oxidative stress in hepatocytes and regulated the AMPK/SIRT1 signaling pathway. (**A**) The lipid content was assessed by Nile Red (red color) and DAPI (blue color) staining. Scale bar = 100 μm. (**B**) Corresponding quantitative analysis of (**A**). (**C**) Intracellular reactive oxygen species (ROS) (green color) assessed by DCFH-DA staining. (**D**) Corresponding quantitative analysis of (**C**). (**E**) Protein expression and/or activation level of SIRT1, AMPK and ACC. (**F**) mRNA expression of lipid metabolism-related genes. *n* = 3–6, ^#^ *p* < 0.05, ^##^ *p* < 0.01 vs. control; * *p* < 0.05, ** *p* < 0.01 vs. model.

To further elucidate the mechanism by which SSF ameliorates MASLD via the AMPK/SIRT1 signaling pathway, in vitro experiments were conducted using PA-induced AML12 hepatocytes incubated with low- or high-dose SSF drug-containing serum or control serum. Nile Red and DAPI co-staining was used to evaluate lipid deposition. Intense red fluorescence was observed in the model group, indicating substantial intracellular lipid accumulation, whereas SSF treatment markedly attenuated lipid deposition in a dose-dependent manner (Figure 6A,B). Intracellular reactive oxygen species (ROS) were assessed by DCFH-DA staining. PA stimulation significantly elevated ROS levels compared with the control group, and this increase was effectively reversed by SSF intervention (Figure 6C,D). Consistent with these findings, Western blotting analysis revealed that the model group exhibited markedly reduced AMPK phosphorylation and SIRT1 protein expression relative to the control group, both of which were dose-dependently restored by SSF treatment. Accordingly, SSF upregulated the phosphorylation of acetyl-CoA carboxylase (ACC), a downstream target of AMPK, and concomitantly decreased the level of acetylated-lysine (Figure 6E). Furthermore, PA treatment significantly downregulated the mRNA expression of key fatty acid oxidation genes, including *Ppargc1α*, *Cpt1α*, and *Acox1*, whereas SSF intervention effectively restored their expression. Conversely, SSF significantly suppressed the PA-induced upregulation of the lipogenic genes *Fasn* and *Srebf1* (Figure 6F).

**Figure 7:**
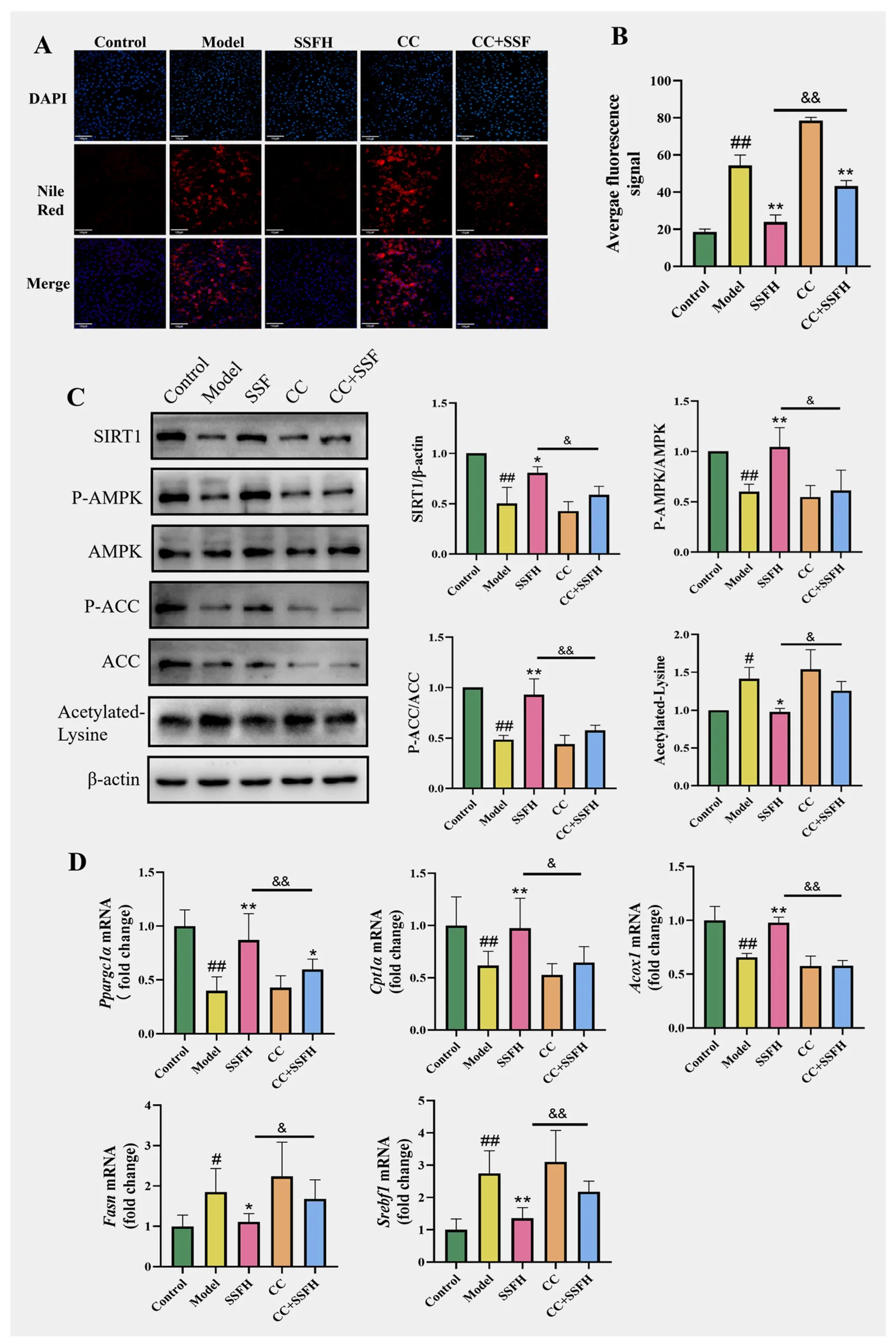
The anti-hepatosteatosis effect of SSF was abrogated by the AMPK inhibitor. (**A**) Lipid content was assessed by Nile Red (red color) and DAPI (blue color) staining. Scale bar = 100 μm. (**B**) Corresponding quantitative analysis of (**A**). (**C**) Protein expression and/or activation of SIRT1, AMPK, ACC and acetylated-lysine. (**D**) mRNA expression levels of lipid metabolism-related genes. *n* = 3–6, ^#^ *p* < 0.05, ^##^ *p* < 0.01 vs. control; * *p* < 0.05, ** *p* < 0.01 vs. model; ^&^ *p* < 0.05, ^&&^ *p* < 0.01 vs. SSFH.

**Figure 8:**
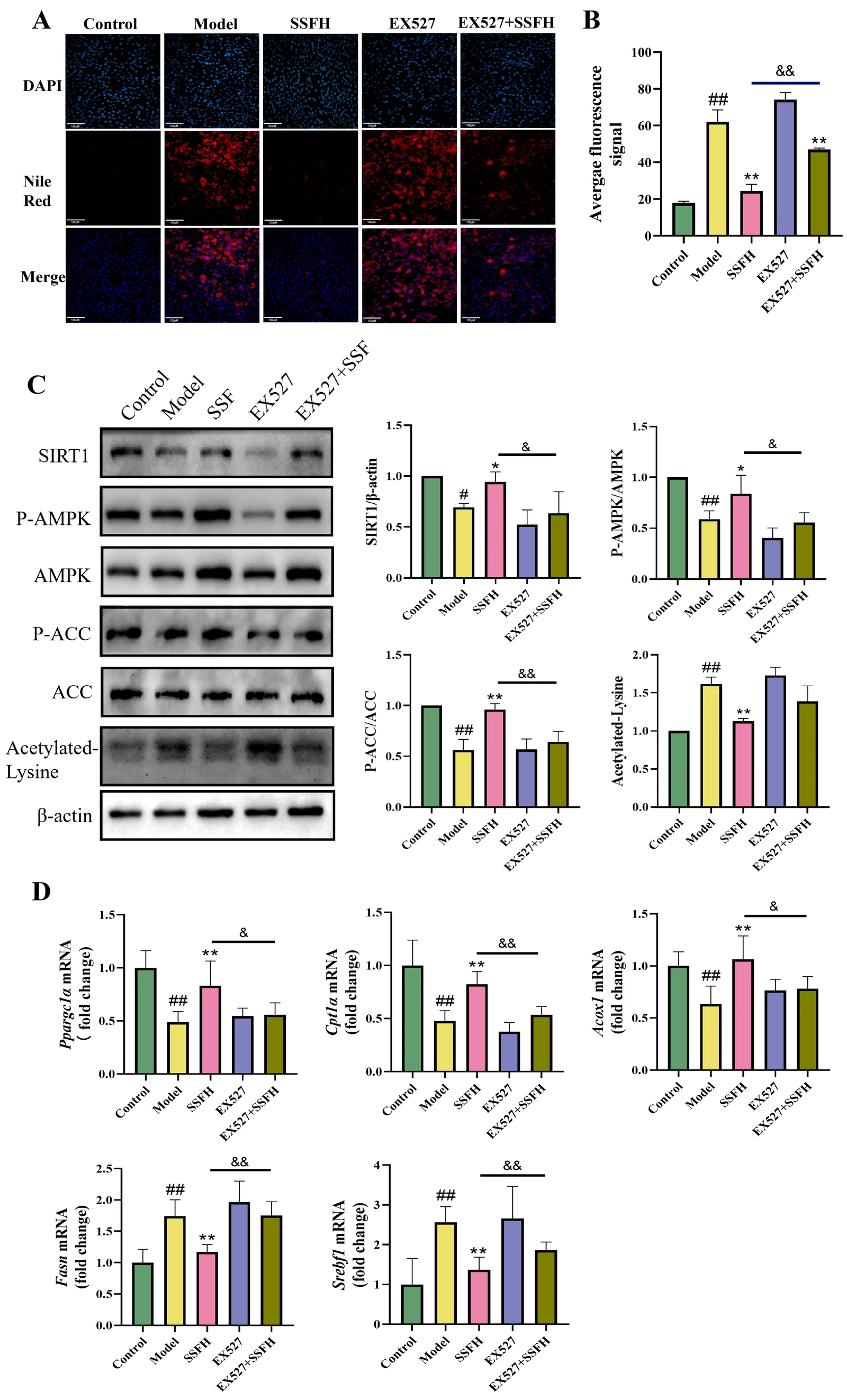
The anti-hepatosteatosis effect of SSF was abrogated by the SIRT1 inhibitor. (**A**) Lipid content was assessed by Nile Red (red color) and DAPI (blue color) staining. Scale bar = 100 μm, the red fluorescence intensity reflects intracellular lipid droplet content. (**B**) Corresponding quantitative analysis of (**A**). (**C**) Protein expression and/or activation of SIRT1, AMPK, ACC and acetylated-lysine. (**D**) mRNA expression levels of lipid metabolism-related genes. *n* = 3–6, ^#^ *p* < 0.05, ^##^ *p* < 0.01 vs. control; * *p* < 0.05, ** *p* < 0.01 vs. model; ^&^ *p* < 0.05, ^&&^ *p* < 0.01 vs. SSFH.

Subsequently, the AMPK inhibitor Compound C or the SIRT1 inhibitor EX527 was applied together with SSF to verify the role of the AMPK/SIRT1 pathway. As shown in Figure 7 and Figure 8, compared with the model group, treatment with Compound C or EX527 led to more pronounced intracellular lipid accumulation in AML12 cells. High-dose SSF (SSFH) significantly mitigated lipid accumulation, whereas this protective effect was largely blocked by co-treatment with Compound C or EX527. Furthermore, compared with the SSFH-treated group, co-incubation of SSFH with Compound C or EX527 significantly decreased the phosphorylation of AMPK and ACC and the expression of SIRT1, and increased the level of acetylated-lysine. Accordingly, the regulatory effect of SSF on the expression of fatty acid oxidation-related genes and lipogenic genes was weakened by both inhibitors.

## 3. Discussion

This study confirmed the therapeutic efficacy of SSF in MASLD model mice induced by an HFD-HF/G diet. Compared with a high-fat diet alone, the addition of liquid fructose and glucose has been shown to exacerbate the severity of MASLD-related phenotypes [22]. Consistently, in our study, mice on the 18-week HFD-HF/G regimen exhibited increased body and liver weights, severe hepatic steatosis, hepatocellular ballooning, and focal inflammatory infiltration with higher MAS scores. In contrast, SSF treatment significantly reduced body and liver weights, alleviated the histopathological damage of liver tissues, and lowered serum levels of lipid and ALT. Notably, berberine, a well-established natural hepatoprotective compound, was used as a positive control. SSF demonstrated superior efficacy to berberine in ameliorating hepatic steatosis, reducing body and liver weights, and improving MAS scores, suggesting that its multi-component nature may confer broader therapeutic benefits through synergistic pathway regulation. Pro-inflammatory cytokines such as TNF-α and IL-6 are positively correlated with MASLD progression and can drive lipid accumulation, insulin resistance, and hepatic inflammation, and accelerate liver fibrosis [23]. ADPN, an endogenous polypeptide secreted by adipocytes, is a multifunctional adipokine that exerts hepatoprotective effects by reducing hepatic lipid biosynthesis, gluconeogenesis, inflammation, and oxidative stress [24]. In this study, elevated serum levels of TNF-α and IL-6, and decreased ADPN levels, were observed in the model group and reversed by SSF intervention. Furthermore, a large number of DEGs were identified among the control, model, and SSF groups through RNA sequencing of mouse liver tissues. The DEGs between the model and SSF groups were primarily enriched in lipid metabolism, while also involving inflammation and fibrosis. Subsequent RT-qPCR validation confirmed that SSF significantly downregulated the expression of genes associated with lipid transport and synthesis, and suppressed expression of genes involved in inflammation and fibrosis.

Additionally, the in vitro MASLD model established using PA-induced AML12 cells also demonstrated the effect of SSF against hepatic lipid accumulation.

Combining our previous network pharmacology predictions—which identified key lipid metabolism targets such as AKT1, SIRT1, and PPARG—with the RNA sequencing pathway enrichment results pointing toward the AMPK signaling pathway, we preliminarily hypothesized that SSF ameliorates lipid metabolic disorders in MASLD by modulating the AMPK/SIRT1 signaling pathway. The aberrant accumulation of hepatic lipids is a critical pathogenic driver of MASLD. Numerous studies have identified the AMPK/SIRT1 axis as a central hub for the regulation of lipid metabolism, the dysregulation of which accelerates MASLD progression.

As a master regulator of energy homeostasis, AMPK is activated in response to an increased intracellular AMP/ATP ratio and subsequently restores energy balance by promoting glucose uptake, fatty acid oxidation (FAO), and ATP production [25]. In HFD-induced MASLD mouse models, downregulation of AMPK phosphorylation is closely linked to the development of macrovesicular steatosis and hepatic inflammation [26]. At the molecular level, activated AMPK inhibits the proteolytic cleavage, maturation, and nuclear translocation of SREBP1c, thereby suppressing its transcriptional activity [27]. Consequently, the expression and activity of FASN, a key downstream lipogenic enzyme, are downregulated, leading to reduced lipid deposition and attenuated inflammation. Furthermore, AMPK directly phosphorylates ACC1/2 at specific serine sites (Ser79/212), neutralizing its catalytic activity and effectively inhibiting de novo lipogenesis (DNL) [28]. This inactivation relieves the inhibition of CPT1α, thereby promoting mitochondrial lipid oxidation [29]. Simultaneously, AMPK activates PGC1α to enhance the transcription of FAO-related genes, upregulating CPT1α and indirectly boosting ACOX1 activity [30]. This coordinated, multi-pathway enhancement of FAO efficiency effectively alleviates hepatic lipid accumulation. Consistent with these findings, our results demonstrated that AMPK phosphorylation was significantly suppressed in the liver tissues of MASLD model mice, accompanied by markedly increased expression of the lipid synthesis-related genes *Srebp1c* and *Fasn*, and significantly decreased expression of the lipid oxidation-related genes *Cpt1α*, *Ppargc1α*, and *Acox1*. Notably, SSF intervention significantly increased AMPK and ACC phosphorylation and reversed the aberrant expression of these downstream targets. In our in vitro experiments, SSF similarly increased AMPK and ACC phosphorylation levels in PA-induced AML12 cells and reversed the abnormal expression of lipid synthesis- and oxidation-related genes, further corroborating the in vivo observations.

SIRT1 is an NAD^+^-dependent deacetylase that consumes NAD^+^ to mediate the deacetylation of histone and non-histone proteins [31], thereby regulating the activity of its substrate proteins by removing specific acetyl-lysine residues [32]. It plays a central role in promoting autophagy to restore mitochondrial function, alleviating oxidative stress, and regulating lipid metabolism-related signaling pathways [33,34]. Previous studies have shown that SIRT1 expression is significantly reduced in patients with MASLD and in animal models, whereas overexpression or activation of SIRT1 can effectively reverse the disease phenotype [35,36]. Enhanced SIRT1 activity inhibits the expression of SREBP1c, whereas SIRT1 knockout upregulates the expression of ChREBP in HepG2 cells [37], further supporting SIRT1 as a potential therapeutic target of considerable value for regulating lipid metabolism in MASLD. In the present study, SSF intervention significantly upregulated SIRT1 expression in both MASLD mouse liver tissues and PA-induced AML12 cells, consistent with its reported protective role.

Importantly, the relationship between AMPK and SIRT1 is not a simple linear upstream–downstream connection but rather a mutually causal, synergistically regulated “metabolic sensing axis”. Within this axis, AMPK activation enhances the deacetylase activity of SIRT1 by increasing intracellular NAD^+^ levels [38], whereas SIRT1 further promotes AMPK phosphorylation through deacetylation of the upstream kinase LKB1 [39]. This bidirectional positive feedback loop significantly inhibits de novo lipogenesis (DNL) mediated by SREBP1 and FASN, reduces lipid droplet accumulation in hepatocytes, and thereby effectively alleviates lipid metabolic disorders [40]. Compound C, an ATP-competitive inhibitor, can block AMPK activation by inhibiting its phosphorylation at the Thr172 site, and EX527 is a highly selective SIRT1 inhibitor that specifically suppresses its deacetylase activity by competitively binding to the nicotinamide-binding site in its catalytic core. Our results demonstrated that the regulatory effects of SSF on AMPK/SIRT1 signaling pathway-related proteins and downstream lipid metabolism-related genes were partially inhibited following intervention by Compound C and EX527. These findings confirm that SSF can ameliorate MASLD, at least in part, by the mechanism of activating the AMPK/SIRT1 signaling pathway to regulate lipid metabolism.

Additionally, AMPK activation can enhance mitochondrial function and promotes Nrf2-mediated antioxidant gene expression [41,42], whereas SIRT1 deacetylates and activates PGC-1α to boost mitochondrial biogenesis and alleviate oxidative damage [43].

The anti-inflammatory and anti-fibrotic effects observed in this study are consistent with the known roles of the AMPK/SIRT1 axis in modulating inflammatory responses and fibrogenesis. AMPK activation has been shown to suppress the NF-κB signaling pathway and inhibit NLRP3 inflammasome activation, thereby reducing the production of pro-inflammatory cytokines such as TNF-α and IL-6 [44]. Similarly, SIRT1 can deacetylate the p65 subunit of NF-κB, attenuating its transcriptional activity and downstream inflammatory cascades [45]. With respect to liver fibrosis, AMPK activation has been reported to inhibit hepatic stellate cell (HSC) activation by suppressing TGF-β/Smad signaling [46], whereas SIRT1 can attenuate fibrogenesis by deacetylating Smad3 and diminishing collagen synthesis [47]. Our transcriptomic analysis identified significant enrichment of inflammation- and fibrosis-related pathways, and RT-qPCR validation confirmed that SSF downregulated the expression of pro-inflammatory genes (*Ccr2*, *Tnf*) and fibrotic markers (*Col1a2*, *Timp1*, *Tgfb2*), supporting the anti-inflammatory and anti-fibrotic potential of SSF through AMPK/SIRT1 activation. Future studies using dedicated fibrosis models are warranted to further characterize these effects. Oxidative stress marked with excessive ROS production is another key pathogenic factor in MASLD, and was driven by excessive fatty acid oxidation (FAO) and mitochondrial dysfunction. DCFH-DA staining confirmed that SSF effectively reversed the PA-induced intracellular ROS elevation in AML12 hepatocytes, suggesting its anti-lipotoxic capacity. To elucidate the material basis of the ameliorative effect of SSF on MASLD, its chemical components were analyzed and identified using UPLC-MS. A total of 77 active compounds were identified, including sinigrin, sinapine, sinapinic acid, chlorogenic acid, caffeic acid, kaempferol, ferulic acid, 4-hydroxy-3-methoxycinnamoylcholine, and luteolin. Some of these compounds have been reported to improve lipid metabolism disorders by regulating the AMPK/SIRT1 signaling pathway. For instance, chlorogenic acid can alleviate palmitic acid-induced lipotoxic damage in hepatocytes by activating SIRT1 and can also achieve multi-dimensional metabolic improvements in T2DM by activating the AMPK signaling pathway [48,49]. Kaempferol has been found to inhibit lipid synthesis and promote FAO by activating the SIRT1/AMPK pathway, thereby alleviating T2DM-associated hepatic lipid deposition [50]. Other studies have also shown that it can activate the AMPK-mediated autophagy pathway, synergistically suppress adipogenesis, enhance FAO, and significantly reduce FFA-induced lipid accumulation in HepG2 cells [51]. Furthermore, rosmarinic acid exerts protective effects by reducing oxidative stress, hepatic lipid accumulation, inflammation, and apoptosis in a mouse MASH model through activation of SIRT1, which subsequently upregulates Nrf2/PPARα and inhibits the NF-κB signaling pathway [52]. Luteolin can ameliorate Western diet-induced atherosclerosis in LDLR-/- mice by activating the AMPK/SIRT1 signaling pathway, thereby inhibiting lipid accumulation and inflammatory responses in macrophages [53]. Collectively, compounds enriched in SSF, such as chlorogenic acid, kaempferol, rosmarinic acid, and luteolin, may collectively target the AMPK/SIRT1 signaling pathway to ameliorate MASLD by alleviating oxidative stress, steatosis, and inflammation, providing a multi-component mechanistic basis for its therapeutic effect. However, although this study confirms the efficacy of SSF in treating MASLD, its specific pharmacodynamic material basis and the synergistic mechanisms among its components remain to be elucidated. Future research will be focused on the high-abundance compounds in SSF. First, candidate compounds that potentially target MASLD pathology can be selected based on literature data. On this basis, in vivo and in vitro experiments will be conducted to systematically evaluate the therapeutic effects of these compounds, either individually or in combination, on MASLD.

## 4. Materials and Methods

### 4.1. Preparation of SSF

Seven TCM herbal materials were used to prepare SSF. *Poria* (batch no. 240902, Anhui, 1-year-old), *Glycyrrhizae Radix et Rhizoma* (batch no. 241213, Gansu, 2-year-old), *Ginseng Radix et Rhizoma* (batch no. 240911, Jilin, 3-year-old), and fried *Raphani Semen* (batch no. 240909, Gansu, 1-year-old) were obtained from Shanghai Hongqiao Chinese Medicine Decoction Pieces Co., Ltd. (Shanghai, China); fried *Sinapis Semen* (batch no. 2024111301, Shandong, 1-year-old) and fried *Perillae Fructus* (batch no. 2024101002, Jiangsu, 1-year-old) were supplied by Shanghai Huaji Pharmaceutical Co., Ltd. (Shanghai, China); and fried *Atractylodis Macrocephalae Rhizoma* (batch no. 20241216-1, Anhui, 1-year-old) was purchased from Shanghai Wanshicheng Guoyao Zhipin Co., Ltd. (Shanghai, China). Morphological, microscopic, and phytochemical identification was performed in accordance with the Chinese Pharmacopoeia (2020 edition). All materials were cleaned and dried at 60 °C to constant weight (moisture content < 12%).

*Ginseng Radix et Rhizoma*, *Atractylodis Macrocephalae Rhizoma*, *Poria*, *Glycyrrhizae Radix et Rhizoma*, *Sinapis Semen*, *Perillae Fructus* and *Raphani Semen* were mixed at a weight ratio of 3:3:3:2:3:3:3, and decocted with water three times. The decoctions were filtered, combined, concentrated and lyophilized at −70 °C under vacuum to obtain a dry extract at a yield of 7.3% (*w*/*w*, dried extract/crude herbs), which was stored at −20 °C until use.

### 4.2. Component Identification and Quantification of Four Components of SSF

The chemical constituents of SSF were analyzed by UPLC-MS. The test solution was prepared by ultrasonic extraction with 50% methanol. Chromatographic separation was performed on a Welch Ultimate UHPLC AQ-C18 column maintained at 35 °C, using a mobile phase consisting of 0.1% formic acid in water (A) and acetonitrile (B). Gradient elution was conducted at a flow rate of 0.3 mL/min, with a DAD detection at 254 nm. Mass spectrometry (MS) was performed on an AB Sciex Triple TOF^®^ 4600 (SCIEX, Framingham, MA, USA) high-resolution mass spectrometer equipped with an electrospray ionization (ESI) source operating in both positive and negative ion modes. The main MS parameters were as follows: both ion source gases 1 and 2, 50 psi; curtain gas, 35 psi; ion source temperature, 500 °C; and ion spray voltage floating, 5000/−4500 V. Data acquisition and processing were performed using Analyst TF 1.7.1 and PeakView 1.2 software, respectively.

Reference standards of sinapine, sinapic acid, ginsenoside Rg1, and glycyrrhizic acid were dissolved in methanol to prepare a mixed reference solution containing 40 μg/mL of each analyte. For the test sample, approximately 0.5 g of SSF powder was accurately weighed, extracted with 10 mL of 50% methanol under ultrasonication for 30 min, and then centrifuged. The supernatant was collected as the sample solution. Chromatographic separation was performed on an Agilent InfinityLab Poroshell 120 AQ-C18 column (2.1 × 100 mm, 2.7 μm) at 35 °C, using a gradient elution with acetonitrile (A) and 0.1% formic acid in water (B) at a flow rate of 0.3 mL/min and an injection volume of 2 μL. Mass spectrometric detection was conducted in positive electrospray ionization (ESI^+^) mode. Quantification was performed using a single-point external standard method based on the peak areas of the corresponding reference standards.

### 4.3. In Vivo Experiment Design

Six-week-old SPF male C57BL/6J mice, weighing 19–21 g, were purchased from Shanghai SLAC Laboratory Animal Co., Ltd. (Shanghai, China), (production license no. SCXK 2022–0004). The mice were housed in the SPF animal facility of Longhua Hospital, Shanghai University of Traditional Chinese Medicine, at 22 ± 2 °C and 55 ± 10% relative humidity, with a 12 h light/dark cycle (light period: 8:00–20:00 daily). After one week of acclimation, the mice were randomly divided, according to body weight, into a control group (*n* = 6) and a model group (*n* = 18). The control group was fed a standard chow diet, whereas the model group was fed an HFD (Research Diets, Inc., New Brunswick, NJ, USA, D12492) combined with high fructose/glucose (HF/G) in the drinking water [54]. From the 11th week onward, the model mice were subdivided into a model group, an SSF group and a berberine group (*n* = 6 per group). The SSF group was treated with SSF by gavage at a daily dose of 574.6 mg/kg and the berberine group was treated with berberine by gavage at a daily dose of 100 mg/kg, whereas the model group received an equivalent volume of normal saline. The SSF dose was converted from the clinically equivalent human dose. At the end of the 18th week, the mice were anesthetized and sacrificed after overnight fasting, and serum and liver tissues were harvested and preserved for subsequent experiments. The animal experimental protocol was approved by the Institutional Animal Ethics Committee of Longhua Hospital, Shanghai University of Traditional Chinese Medicine (No. LHERAW-23045).

### 4.4. Serum Biochemical Parameter Analysis

Serum levels of TC, TG, LDL-c, and ALT were quantified using corresponding assay kits (Jiancheng Bioengineering Institute, Nanjing, China) and a Synergy H4 Microplate Reader (BioTek, Winooski, VT, USA) [55].

### 4.5. Enzyme-Linked Immunosorbent Assay (ELISA)

Serum levels of tumor necrosis factor-α (TNF-α), interleukin-6 (IL-6) and adiponectin (ADPN) were determined using enzyme-linked immunosorbent assay kits (Shanghai Enzyme-linked Biotechnology Co., Ltd., Shanghai, China) according to the manufacturers’ instructions [56].

### 4.6. Histopathology of Liver Tissues

Mice liver tissue samples were fixed in 4% paraformaldehyde. After dehydration, the tissues were paraffin-embedded and cut into 5 μm sections for hematoxylin and eosin (H&E) staining. The H&E-stained sections were photographed under a light microscope and subjected to the MASLD Activity Score (MAS) evaluation, which comprises three components: steatosis (0–3), lobular inflammation (0–2) and hepatocellular ballooning (0–2). A total MAS score ≥ 5 indicates a diagnosis of MASH [57].

### 4.7. Oil Red O Staining

For frozen section analysis, liver tissues were snap-frozen, embedded in optimal cutting temperature (OCT) compound, and sectioned into 10 μm frozen slices. The slices were stained with Oil Red O solution, rinsed with 75% ethanol, and counterstained with hematoxylin for nuclear labeling. The sections were then mounted with 85% glycerol, and photographed under a light microscope for morphological analysis.

### 4.8. Preparation of SSF Drug-Containing Serum

Sprague-Dawley rats were randomly divided, according to body weight, into an SSF group and a control group (*n* = 15 per group). Rats in the SSF group were given SSF by gavage at a daily dose of 460.4 mg/kg, whereas the control group received an equal volume of normal saline, twice daily for 5 consecutive days. One hour after the last administration, the rats were anesthetized with 2% sodium pentobarbital (50 mg/kg) by intraperitoneal injection (i.p.). Blood was collected from the abdominal aorta and left at room temperature for 2 h. After inactivation and filtration for sterilization, the serum was stored at −80 °C [58].

### 4.9. In Vitro Experiment Design

AML12 cells were purchased from the Cell Bank of the Chinese Academy of Sciences (Shanghai, China). The cells were cultured in high-glucose DMEM medium supplemented with 10% fetal bovine serum (FBS), 100 U/mL penicillin and 100 μg/mL streptomycin in a humidified incubator at 37 °C with 5% CO_2_. The in vitro MASLD model was established by inducing AML12 cells with 0.5 mM PA and 1% BSA (Sigma, Steinheim, Germany) for 24 h [59]. Simultaneously, the cells were treated with various doses of SSF drug-containing serum, 1 μM Compound C (AMPK inhibitor) or 10 μM EX527 (SIRT1 inhibitor) (MedChemExpress, Shanghai, China). Cells cultured in 1% BSA-DMEM served as the control.

### 4.10. Nile Red Staining

The cells were fixed with 4% paraformaldehyde and then stained with Nile Red staining solution (Sigma, St. Louis, MO, USA) for 15 min. After washing with phosphate-buffered saline (PBS), the cells were incubated with DAPI staining solution (YEASEN, Shanghai, China) at room temperature for 10 min. The cells were observed and photographed under a fluorescence microscope, and the fluorescence intensity was quantified using ImageJ software (version 1.54, NIH, Bethesda, MD, USA).

### 4.11. Detection of Intracellular Reactive Oxygen Species

Intracellular reactive oxygen species (ROS) were detected using a ROS assay kit (Beyotime, Shanghai, China) containing the fluorescent probe DCFH-DA. The cells were washed twice with PBS, incubated with the diluted DCFH-DA (1:1000) at 37 °C for 20 min, and washed twice again. The cell nuclei were then counterstained with Hoechst 33342 (Beyotime) at 37 °C for 10 min. After three additional washes, the cells were immediately observed and photographed under a fluorescence microscope.

### 4.12. RNA Sequence Analysis

Total RNA was isolated from the samples using the RNeasy Mini Kit (Qiagen, Hilden, Germany), and paired-end libraries were prepared with the TruSeq^®^ RNA Sample Preparation Kit (Illumina, San Diego, CA, USA) according to the manufacturer’s guide: poly-A mRNA was purified using poly-T oligo-attached magnetic beads, followed by quantification with a Qubit^®^ 2.0 Fluorometer (Life Technologies, Carlsbad, CA, USA) and validation of insert size and molar concentration using an Agilent 2100 Bioanalyzer (Agilent Technologies, Santa Clara, CA, USA). Libraries (10 pM) were clustered on a cBot and sequenced on an Illumina Novaseq 6000 (Illumina, Austin, TX, USA) by Shanghai Biotechnology Corporation. Raw reads were preprocessed to remove rRNA, adapters, short fragments, and low-quality reads; the cleaned reads were mapped to the mouse GRCm38 genome (2 mismatches allowed) using Hisat2 (v2.0.4) [60]. Fragments per kilobase of transcript per million mapped reads (FPKM) values were calculated with Stringtie (v1.3.0) [61,62], and differentially expressed genes (FDR ≤ 0.05, fold-change ≥ 2) were identified using edgeR (v4.11.4) [63].

### 4.13. Reverse Transcription-Quantitative PCR (RT-qPCR)

Total RNA was extracted from liver tissues or cells using Trizol reagent (Ambion, Austin, TX, USA). cDNA synthesized was performed with a reverse transcription kit (Accurate Biology, Changsha, China). Gene-specific transcripts were amplified by quantitative PCR (qPCR) on a StepOnePlus Real-Time PCR System (Applied Biosystems, Foster, CA, USA) using an SYBR Green PCR Mix (Accurate Biology, Changsha, China). β-actin was used as the internal reference gene. Primers for the mouse target genes were designed and synthesized by Shanghai Shinegene Biotechnology Co., Ltd. (Shanghai, China); the primer sequences are provided in Table 2. Relative mRNA expression levels were calculated using the 2−ΔΔCT method. All qPCR experiments were performed in accordance with the MIQE guidelines [64]. Table 3 lists the PCR primer sequences utilized in this study.

### 4.14. Western Blot

Proteins were extracted from liver tissues or cells using RIPA lysis buffer (Beyotime Biotechnology, Shanghai, China) freshly supplemented with phosphatase and protease inhibitors (Millipore, Billerica, MA, USA). Protein concentrations were quantified using a BCA protein assay kit (Beyotime Biotechnology). Proteins were then separated by 10% sodium dodecyl sulfate–polyacrylamide gel electrophoresis (SDS-PAGE) and transferred to polyvinylidene fluoride (PVDF) membranes (Millipore). After blocking, the membranes were incubated with primary antibodies against SIRT1 (Abcam, Cambridge, MA, USA), P-AMPK, acetyl-CoA carboxylase (ACC), P-ACC, and acetylated-lysine (Cell Signaling Technology, Danvers, MA, USA), and AMPK and β-actin (Abclonal, Wuhan, China) at 4 °C overnight. After incubation with secondary antibodies (Abclonal) at room temperature for 1 h, protein band signals were developed using an Enhanced Chemiluminescence (ECL) Detection Kit (Millipore) and captured on a Tanon 5200 imaging system (Tanon, Shanghai, China). Band intensities were quantified using ImageJ software, and protein expression levels were normalized to β-actin.

### 4.15. Statistical Analysis

Statistical analyses and graphing were performed using SPSS 25.0 and GraphPad Prism 8.0. Quantitative data are expressed as the mean ± SD. One-way analysis of variance (ANOVA) was used for comparisons among multiple groups, followed by Dunnett’s post hoc test. A *p*-value < 0.05 was considered statistically significant.

## 5. Conclusions

**Figure 9:**
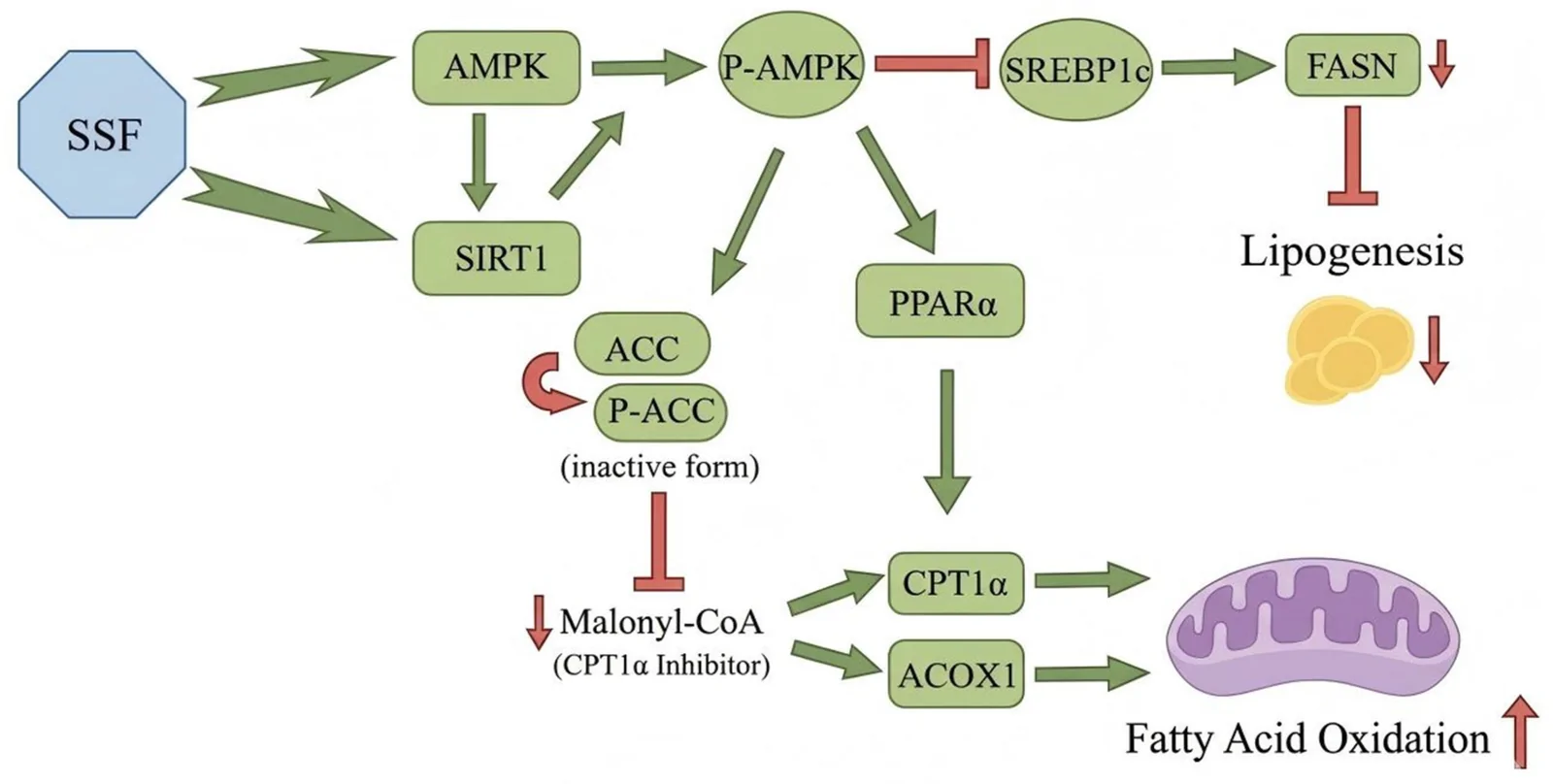
Schematic mechanism of SSF in alleviating MASLD via the AMPK/SIRT1 pathway.

In summary, the present study validated the therapeutic potential of SSF against diet-induced MASLD in mice, as evidenced by improvements in liver histopathology and function, and systemic metabolic disorders. UPLC-MS analysis identified 77 bioactive components in SSF, several of which have been previously reported to regulate lipid metabolism via the AMPK/SIRT1 axis. Further in vivo and in vitro experiments revealed that SSF exerts protective effects mainly by activating the AMPK/SIRT1 signaling cascade, thereby improving lipid metabolic dysregulation and mitigating lipotoxic liver injury (Figure 9). These results offer experimental evidence supporting the clinical use of SSF for MASLD treatment and may facilitate the development of novel TCM component-based drugs. Future studies are required to select and verify the key bioactive components contributing to the efficacy of SSF, and elucidate their individual and synergistic effects in MASLD.

## Figures and Tables

**Table 1:** Active components of SSF by UPLC-MS analysis.

NO.	Time (min)	Polarity	*m*/*z*	ppm	Formula	Name	Area Percentage	Attribution
1	3.36	[M−H]^−^	358.0272	0.0	C_10_H_17_NO_9_S_2_	Sinigrin	12.602%	A/B
2	3.40	[M+H]^+^	268.1039	−0.4	C_10_H_13_N_5_O_4_	Adenosine	0.160%	all
3	3.82	[M−H]^−^	434.0264	3.2	C_12_H_21_NO_10_S_3_	Glucoraphenin	29.261%	A/B
4	4.19	[M+H]^+^	284.0986	−1.1	C_10_H_13_N_5_O_5_	Guanosine	0.059%	all
5	6.91	[M+H]^+^	205.0962	−4.7	C_11_H_12_N_2_O_2_	L-tryptophan	0.050%	A/B
6	8.98	[M−H]^−^	353.0897	5.4	C_16_H_18_O_9_	Neochlorogenic acid	0.044%	F
7	9.88	[M−H]^−^	209.0464	4.3	C_10_H_10_O_5_	(p-Hydroxybenzyl)malonic acid	0.174%	all
8	10.22	M^+^	280.1534	−0.8	C_15_H_22_NO_4_^+^	4-Hydroxy-3-methoxycinnamoylcholine	0.618%	A/B
9	10.23	[M+FA−H]^−^	493.2309	3.6	C_21_H_36_O_10_	Atractyloside A	0.112%	F
10	10.91	M^+^	310.1661	3.9	C_16_H_24_NO_5_^+^	Sinapine	12.318%	A/B
11	11.59	[M−H]^−^	353.0877	−0.3	C_16_H_18_O_9_	Chlorogenic acid	0.055%	F
12	11.85	[M−H]^−^	179.0359	5.0	C_9_H_8_O_4_	Caffeic acid	0.052%	C
13	12.22	[M−H]^−^	353.0876	−0.6	C_16_H_18_O_9_	Cryptochlorogenic acid	0.041%	F
14	12.63	M^+^	476.2264	−3.1	C_25_H_34_NO_8_^+^	2-[[3-[4-[2-Hydroxy-2-(4-hydroxy-3-methoxyphenyl)-1-(hydroxymethyl)ethoxy]-3-methoxyphenyl]-1-oxo-2-propen-1-yl]oxy]-N,N,N-trimethylethanaminium	1.146%	A/B
15	14.18	[M−H]^−^	415.1043	1.9	C_21_H_20_O_9_	Puerarin	2.875%	/
16	14.63	[M−H]^−^	977.2622	5.5	C_44_H_50_O_25_	Kaempferol 3-sinapoylsophoroside-7-O-glucoside	0.174%	A/B
17	15.08	M^+^	506.2377	−1.6	C_26_H_36_NO_9_^+^	2-[[(2E)-3-[4-[2-Hydroxy-2-(4-hydroxy-3-methoxyphenyl)-1-(hydroxymethyl)ethoxy]-3,5-dimethoxyphenyl]-1-oxo-2-propen-1-yl]oxy]-N,N,N-trimethylethanaminium	0.636%	A/B
18	16.69	M^+^	506.2371	−2.8	C_26_H_36_NO_9_^+^	2-[[3-[4-[2-Hydroxy-2-(4-hydroxy-3-methoxyphenyl)-1-(hydroxymethyl)ethoxy]-3,5-dimethoxyphenyl]-1-oxo-2-propen-1-yl]oxy]-N,N,N-trimethylethanaminium	0.704%	A/B
19	17.00	[M−H]^−^	193.0507	0.5	C_10_H_10_O_4_	Ferulic acid	0.045%	C
20	17.80	[M−H]^−^	223.0629	7.6	C_11_H_12_O_5_	Sinapic acid	0.476%	A/B
21	18.31	M^+^	458.2184	2.4	C_25_H_32_NO_7_^+^	2-[[3-[2,3-Dihydro-2-(4-hydroxy-3-methoxyphenyl)-3-(hydroxymethyl)-7-methoxy-5-benzofuranyl]-1-oxo-2-propen-1-yl]oxy]-N,N,N-trimethylethanaminium	0.463%	A/B
22	18.52	[M−H]^−^	417.1200	2.2	C_21_H_22_O_9_	Liquiritin	1.923%	G
23	18.83	[M−H]^−^	549.1636	4.0	C_26_H_30_O_13_	Liquiritin apioside	2.202%	G
24	19.26	[M−H]^−^	753.2294	6.1	C_34_H_42_O_19_	1,2-Disinapoylgentiobiose	0.134%	A/B
25	19.66	[M−H]^−^	753.2279	4.1	C_34_H_42_O_19_	1,4-Disinapoylgentiobiose	0.193%	A/B
26	20.18	[M−H]^−^	521.1321	3.8	C_24_H_26_O_13_	Salviaflaside	1.154%	C
27	20.49	[M−H]^−^	187.0978	1.1	C_9_H_16_O_4_	Azelaic acid	0.195%	C
28	21.73	[M−H]^−^	753.2283	4.6	C_34_H_42_O_19_	3′,6-Disinapoylsucrose	0.443%	A/B
29	21.75	[M−H]^−^	640.0886	8.9	C_23_H_31_NO_14_S_3_	[3,4,5-trihydroxy-6-[(E)-C-[(E)-4-methylsulinylbut-3-enyl]-N-sulooxycarbonimidoyl]sulanyloxan-2-yl]methyl(E)-3-(4-hydroxy-3,5-dimethoxyphenyl)prop-2-enoate	8.853%	A/B
30	22.03	[M−H]^−^	359.0793	5.8	C_18_H_16_O_8_	Rosmarinic acid	0.873%	C
31	22.75	[M+FA−H]^−^	475.1271	5.3	C_22_H_22_O_9_	Ononin	0.275%	G
32	22.85	M^+^	682.2872	2.1	C_36_H_44_NO_12_^+^	2-[[3-[4-[2-Hydroxy-1-[hydroxy(4-hydroxy-3-methoxyphenyl)methyl]ethoxy]-3-methoxyphenyl]-1-oxo-2-propen-1-yl]oxy]-N,N,N-trimethylethanaminium mono [3-(4-hydroxy-3,5-dimethoxyphenyl)-2-propenoate] (ester)	0.499%	A/B
33	22.89	[M−H]^−^	549.1650	6.6	C_26_H_30_O_13_	Isoliquiritin apioside	0.275%	G
34	23.04	M^+^	712.2971	1.0	C_37_H_46_NO_13_^+^	2-[[3-[4-[2-Hydroxy-1-[hydroxy(4-hydroxy-3-methoxyphenyl)methyl]ethoxy]-3,5-dimethoxyphenyl]-1-oxo-2-propen-1-yl]oxy]-N,N,N-trimethylethanaminium mono [3-(4-hydroxy-3,5-dimethoxyphenyl)-2-propenoate] (ester)	0.229%	A/B
35	23.23	M^+^	712.2969	0.7	C_37_H_46_NO_13_^+^	2-[[3-[4-[2-Hydroxy-2-(4-hydroxy-3,5-dimethoxyphenyl)-1-(hydroxymethyl)ethoxy]-3-methoxyphenyl]-1-oxo-2-propen-1-yl]oxy]-N,N,N-trimethylethanaminium mono [3-(4-hydroxy-3,5-dimethoxyphenyl)-2-propenoate] (ester)	0.630%	A/B
36	23.24	[M−H]^−^	417.1223	7.7	C_21_H_22_O_9_	Isoliquiritin	0.450%	G
37	23.56	[M−H]^−^	445.0802	5.8	C_21_H_18_O_11_	Apigenin 7-O-β-D-glucuronide	4.427%	C
38	24.00	[M+FA−H]^−^	845.495	5.4	C_42_H_72_O_14_	Ginsenoside Rg1	0.987%	D
39	24.06	[M+FA−H]^−^	991.5546	6.4	C_48_H_82_O_18_	Ginsenoside Re	0.279%	D
40	25.00	[M−H]^−^	285.0399	−2.1	C_15_H_10_O_6_	Luteolin	0.452%	C
41	26.20	[M−H]^−^	459.0954	4.6	C_22_H_20_O_11_	Acacetin 7-O-glucuronide	0.657%	C
42	27.08	[M−H]^−^	269.0459	1.5	C_15_H_10_O_5_	Apigenin	0.226%	C
43	27.36	[M−H]^−^	853.3903	5.3	C_42_H_62_O_18_	22-Hydroxy-licorice saponin G2	0.187%	G
44	27.40	[M+FA−H]^−^	845.4977	8.6	C_42_H_72_O_14_	Ginsenoside Rf	0.312%	D
45	27.67	[M−H]^−^	983.4581	8.9	C_48_H_72_O_21_	Licorice saponin A3	0.403%	G
46	27.80	[M+FA−H]^−^	815.4866	8.3	C_41_H_70_O_13_	Ginsenoside F5	0.167%	D
47	28.13	[M+FA−H]^−^	1153.6091	6.9	C_54_H_92_O_23_	Ginsenoside Rb1	0.175%	D
48	28.17	[M+FA−H]^−^	829.4964	1.1	C_42_H_72_O_13_	20(S)-Ginsenoside Rg2	0.136%	D
49	28.21	[M−H]^−^	879.4088	7.7	C_44_H_64_O_18_	22β-acetoxyl-glycyrrhizin	0.244%	G
50	28.24	[M−H]^−^	837.3982	8.1	C_42_H_62_O_17_	Licoricesaponin G2	0.532%	G
51	28.28	[M+FA−H]^−^	829.5021	8.0	C_42_H_72_O_13_	20(R)-Ginsenoside Rg2	0.074%	D
52	28.40	[M+FA−H]^−^	1123.6003	8.6	C_53_H_90_O_22_	Ginsenoside Rc	0.114%	D
53	28.49	[M+FA−H]^−^	683.4427	7.5	C_36_H_62_O_9_	Ginsenoside Rh1	0.308%	D
54	28.50	[M−H]^−^	255.0671	3.1	C_15_H_12_O_4_	Liquiritigenin	0.029%	G
55	28.51	[M−H]^−^	267.0675	4.5	C_16_H_12_O_4_	Formononetin	0.039%	G
56	28.61	[M+FA−H]^−^	1123.5988	7.3	C_53_H_90_O_22_	Ginsenoside Rb2	0.125%	D
57	28.87	[M+FA−H]^−^	1195.6227	9.2	C_56_H_94_O_24_	Quinquenoside R1	0.018%	D
58	29.17	[M+FA−H]^−^	991.5517	3.4	C_48_H_82_O_18_	Ginsenoside Rd	0.098%	D
59	29.22	[M−H]^−^	837.3911	−0.4	C_42_H_62_O_17_	Uralsaponin U	0.780%	G
60	29.39	[M−H]^−^	837.3911	−0.4	C_42_H_62_O_17_	Uralsaponin N	0.212%	G
61	29.77	[M−H]^−^	821.4020	6.7	C_42_H_62_O_16_	Glycyrrhizic acid	3.884%	G
62	30.36	[M−H]^−^	821.4034	8.4	C_42_H_62_O_16_	Uralsaponin B	1.813%	G
63	30.59	[M−H_2_O+H]^+^	231.1391	4.8	C_15_H_20_O_3_	Atractylenolide III	0.043%	F
64	30.79	[M−H]^−^	367.1184	−0.9	C_21_H_20_O_6_	Glycycoumarin	0.258%	G
65	31.52	[M−H]^−^	807.4234	7.7	C_42_H_64_O_15_	Licoricesaponin B2	0.104%	G
66	31.52	[M−H]^−^	353.1033	0.6	C_20_H_18_O_6_	Licoisoflavone A	0.400%	G
67	31.68	[M+FA−H]^−^	829.5001	5.5	C_42_H_72_O_13_	20(S)-Ginsenoside Rg3	0.331%	D
68	31.82	[M+FA−H]^−^	829.5006	6.1	C_42_H_72_O_13_	20(R)-Ginsenoside Rg3	0.115%	D
69	31.86	[M−H]^−^	497.3261	−2.3	C_31_H_46_O_5_	6α-Hydroxypolyporenic acid C	0.023%	E
70	32.60	[M+H]^+^	233.1541	2.1	C_15_H_20_O_2_	Atractylenolide II	0.020%	F
71	32.71	[M−H]^−^	351.0881	2.0	C_20_H_16_O_6_	Licoisoflavone B	0.246%	G
72	33.18	[M+FA−H]^−^	811.4927	9.6	C_42_H_70_O_12_	Ginsenoside Rk1	0.093%	D
73	33.32	[M+FA−H]^−^	811.4915	8.1	C_42_H_70_O_12_	Ginsenoside Rg5	0.098%	D
74	33.42	[M−H]^−^	483.3141	3.3	C_30_H_44_O_5_	Poricoic acid B	0.018%	E
75	33.84	[M−H]^−^	497.3291	6.3	C_31_H_46_O_5_	Poricoic acid A	0.017%	E
76	33.92	[M−H]^−^	485.3293	4.3	C_30_H_46_O_5_	Poricoic acid G	0.010%	E
77	34.28	[M−H]^−^	481.3357	1.2	C_31_H_46_O_4_	Polyporenic acid C	0.004%	E
78	35.73	[M+H]^+^	256.2643	3.1	C_16_H_33_NO	Hexadecanamide	0.305%	H
79	35.94	[M+H]^+^	282.2805	5.0	C_18_H_35_NO	Oleamide	0.843%	H

Abbreviations: A, Sinapis Semen; B, Raphani Semen; C, Perillae Fructus; D, Ginseng Radix et Rhizoma; E, Poria; F, Atractylodis Macrocephalae Rhizoma; G, Glycyrrhizae Radix et Rhizoma; H, plastic-derived artifact.

**Table 2:** Quantitative determination of four components in SSF by LC-MS/MS.

Compounds	Content (mg/g SSF)
Sinapine	3.87
Sinapic acid	2.00
Ginsenoside Rg1	0.76
Glycyrrhizic acid	1.82

**Table 3:** RT-qPCR primer sequences.

Protein Name	Gene Name	Primer Sequence
Beta-actin (β-actin)	beta-actin (*Actb*)	Forward: GAGACCTTCAACACCCCAGC Reverse: ATGTCACGCACGATTTCCC
Cluster of differentiation 36 (CD36)	cluster of differentiation 36 (*Cd36*)	Forward: CAACATATTGGTCAAGCCAGC Reverse: AGTAAGGCCATCTCTACCATGC
Stearoyl-CoA desaturase 1 (SCD1)	stearoyl-CoA desaturase 1 (*Scd1*)	Forward: ATTGCCTATGAACTCAACAGCG Reverse: TGCCATAGTGGTTGAGGTTGG
Tumor necrosis factor-alpha (TNF-α)	tumor necrosis factor alpha (*Tnf*)	Forward: CCCTCCAGAAAAGACACCATG Reverse: CACCCCGAAGTTCAGTAGACAG
Fatty acid-binding protein 4 (FABP4)	fatty acid binding protein 4 (*Fabp4*)	Forward: CAGACGACAGGAAGGTGAAGAG Reverse: TTTATTGTGGTCGACTTTCCATC
C-C chemokine receptor type 2 (CCR2)	C-C chemokine receptor type 2 (*Ccr2*)	Forward: CTGCCTCCACTCTACTCCCTG Reverse: GACCCACTCATTTGCAGCATAG
Collagen type I alpha 2 chain (COL1α2)	collagen type I alpha 2 chain (*Col1a2*)	Forward: TTGCAGTAACTTCGTGCCTAGC Reverse: TGGGACCATCAACACCATCTC
Tissue inhibitor of metalloproteinases 1 (TIMP1)	TIMP metallopeptidase inhibitor 1 (*Timp1*)	Forward: GCCCACAAGTCCCAGAACC Reverse: TACGCCAGGGAACCAAGAAG
Transforming growth factor-beta 2 (TGF-β2)	transforming growth factor beta 2 (*Tgfb2*)	Forward: TCGACATGGATCAGTTTATGCG Reverse: CCCTGGTACTGTTGTAGATGGA
Peroxisome proliferator-activated receptor gamma coactivator 1-alpha (PGC-1α)	PPAR gamma coactivator 1 alpha (*Ppargc1α*)	Forward: TGGCACGCAGCCCTATTC Reverse: GAGGATCTACTGCCTGGGGAC
Carnitine palmitoyltransferase 1 alpha (CPT1α)	carnitine palmitoyltransferase 1 alpha (*Cpt1α*)	Forward: GCCATACTGCTGTATCGTCGC Reverse: CGGGAAGTATTGAAGAGTCGC
Acyl-CoA oxidase 1 (ACOX1)	acyl-CoA oxidase 1 (*Acox1*)	Forward: CTCGGAAGATACATAAAGGAGACC Reverse: CCAGGTAGTAAAAGCCTTCAGC
Fatty acid synthase (FASN)	fatty acid synthase (*Fasn*)	Forward: TGGGCTGTCCTGCCTCTG Reverse: GTTTCACGAACCCGCCTC
Sterol regulatory element-binding protein 1c (SREBP1c)	sterol regulatory element binding transcription factor 1 (*Srebf1*)	Forward: ACAAAAGCAAATCACTGAAGGAC Reverse: GGGCTCAGAGTCACTACCACC

## Data Availability

The datasets used and/or analyzed during the current study are available from the corresponding author on reasonable request.

## Supplementary Materials

The following supporting information can be downloaded at: https://www.mdpi.com/article/10.3390/ph19081195/s1, Figure S1: LC-MS/MS quantification of four components in SSF.
