# Supplementary material for: Sanzi Sijun Formula Alleviates Lipotoxic Liver Injury in Metabolic Dysfunction-Associated Steatotic Liver Disease via AMPK/SIRT1 Signaling Pathway
Source: Pharmaceuticals (Basel). 2026 Jul 29;19(8):1195. doi: 10.3390/ph19081195 (PMC13516140; doi:10.3390/ph19081195)
Supplement: Supplementary file 1 [file pharmaceuticals-19-01195-s001.zip › pharmaceuticals-4311547-supplementary.pdf]

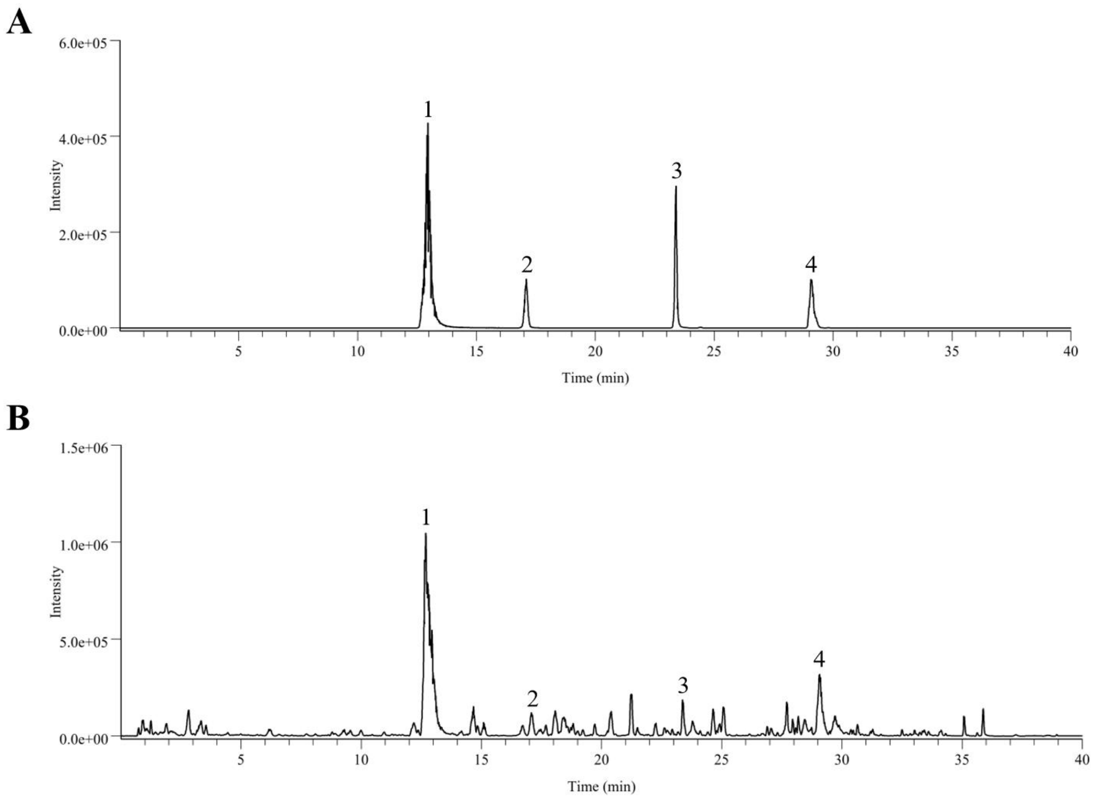


**Figure S1.** LC-MS/MS quantification of four components in SSF. Representative LC-MS/MS chromatograms in positive ion mode: (A) reference standards; (B) SSF sample. Peaks: 1, sinapine; 2, sinapic acid; 3, ginsenoside Rg1; 4, glycyrrhizic acid.
